# Major hepatic resection may suppress the growth of tumours remaining in the residual liver

**DOI:** 10.1054/bjoc.2000.1379

**Published:** 2000-10

**Authors:** H Yokoyama, S Goto, C-L Chen, T-L Pan, K Kawano, S Kitano

**Affiliations:** Department of Surgery, Chang Gung Memorial Hospital, 123, Ta-Pei Rd., Niao-Sung, Kaohsiung, Taiwan; Department of Surgery I, Oita Medical University, Hasama-machi, Oita, 879–5593, Japan

**Keywords:** HCC, hepatectomy, tumour growth, apoptosis, rat

## Abstract

Little is known as to how hepatectomy is associated with the growth of hepatic tumours, which may reside in the remaining liver after curative resection for hepatocellular carcinoma. Using an intra-hepatic tumour implantation model in rats, the effects of hepatectomy on tumour growth in the remaining liver were investigated. On post-operative day 7, the tumour weight in the remaining liver following 30% hepatectomy was 0.321 ± 0.058 g (mean ± SD) which was significantly greater than that (0.245 ± 0.040 g) in sham operations (*P* < 0.05). However, the tumour weight (0.156 ± 0.067 g) in the remaining liver following 60% hepatectomy was significantly lower than that in sham animals (*P* < 0.005). The number of TdT-mediated dUTP nick-end labelling (TUNEL) positive tumour cells was significantly increased in 60% hepatectomy as compared with the sham and 30% hepatectomy group. The mRNA expression of TGF-β1, TNF-α and Fas in the tumour portion of 60% hepatectomy, was higher than that in 30% hepatectomy group. Plasma levels of TGF-β1 were inversely correlated with intra-hepatic tumour weights. These results suggest that major hepatic resection may lead to an increased induction of apoptosis for the remaining hepatic tumour. © 2000 Cancer Research Campaign


*British Journal of Cancer* (2000) **83**(8), 1096–1101
© 2000 Cancer Research Campaign
doi: 10.1054/ bjoc.2000.1379, available online at http://www.idealibrary.com on IDEAL®

### Major hepatic resection may suppress the growth of tumours remaining in the residual liver

**H Yokoyama*†, S Goto*†, C-L Chen*, T-L Pan*, K Kawano† and S Kitano†**

*Department of Surgery, Chang Gung Memorial Hospital, 123, Ta-Pei Rd., Niao-Sung, Kaohsiung, Taiwan and †Department of Surgery I, Oita Medical University, Hasama-machi, Oita, 879–5593, Japan

**Summary** Little is known as to how hepatectomy is associated with the growth of hepatic tumours, which may reside in the remaining liver after curative resection for hepatocellular carcinoma. Using an intra-hepatic tumour implantation model in rats, the effects of hepatectomy on tumour growth in the remaining liver were investigated. On post-operative day 7, the tumour weight in the remaining liver following 30% hepatectomy was 0.321 ± 0.058 g (mean ± SD) which was significantly greater than that (0.245 ± 0.040 g) in sham operations ($P < 0.05$). However, the tumour weight (0.156 ± 0.067 g) in the remaining liver following 60% hepatectomy was significantly lower than that in sham animals ($P < 0.005$). The number of TdT-mediated dUTP nick-end labelling (TUNEL) positive tumour cells was significantly increased in 60% hepatectomy as compared with the sham and 30% hepatectomy group. The mRNA expression of TGF-β1, TNF-α and Fas in the tumour portion of 60% hepatectomy, was higher than that in 30% hepatectomy group. Plasma levels of TGF-β1 were inversely correlated with intra-hepatic tumour weights. These results suggest that major hepatic resection may lead to an increased induction of apoptosis for the remaining hepatic tumour. © 2000 Cancer Research Campaign

**Keywords:** HCC; hepatectomy; tumour growth; apoptosis; rat

Liver resection for hepatocellular carcinoma (HCC), which is one of the most common malignant tumours, is still considered as the most radical treatment. Recent advances in surgical techniques for hepatectomy have made surgical intervention a safe and effective treatment for HCC. However, even if the resection is curative, recurrences in the remaining liver frequently occur (Nagao et al, 1990; Yamamoto et al, 1996). Once such recurrences are found, the patient prognosis is poor. In reality, the overall 5 year survival rate of patients with HCC after curative resection has been reported to exceed fifty per cent (Kosuge et al, 1993). Therefore, the prevention of recurrences is important for patients with HCC, however the mechanisms of such recurrences remain unclear.

When HCC patients are operated upon, intra-operative manipulation should be as careful as possible in order not to mechanically disperse any possible cancer cells (Ezaki et al, 1989). Despite such careful manipulation, residual microscopic HCC cells undetected by pre-operative imaging for diagnosis, can cause recurrences (Utsunomiya et al, 1992). Other investigators have shown that most recurrent tumours occurred predominantly in the remaining liver near the primary lesion rather than in distant segments (Nagao et al, 1990; Yamamoto et al, 1996). Further, most secondary tumours in the remaining liver after the first hepatic resection were considered to be caused by metastatic recurrence and not by secondary carcinogenesis, the so-called ‘multicentric occurrence’ (Shimada et al, 1998). These reports allow us to speculate that the most likely causes of HCC recurrence may be due to: (a) the cancer cells dispersed by intra-operative manipulation and (b) existence of HCC in the remaining liver following curative resection, which is the established tumour undetected pre-operatively.

Several experiments for acceleration of intra-hepatic tumour growth by partial hepatectomy in animal models have been trying to prove how cancer cells are distributed in the liver following hepatectomy in conjunction with the above explanation (a) (Mizutani et al, 1992; Slooter et al, 1995; Picardo et al, 1998). However, it is still controversial as to whether partial hepatic resection renders progression or regression of tumour cells (Ono et al, 1986; Castillo et al, 1989). The different timing of hepatectomy before or after administration of different tumour cell lines, via different routes, may cause inconsistent results for these experimental studies. Additionally, very few studies have reported the effect of hepatectomy on established HCC in the residual liver as outlined above in point (b). In the present study using an intra-hepatic tumour implantation model, the effects of hepatic resection on the growth of the established tumour in the remaining liver were investigated and discussed from the aspect of apoptosis.

### MATERIALS AND METHODS

### Animals

Adult male Donryu rats (Charles River Japan, Inc., Yokohama, Japan), 6–7 weeks old, weighing 170–245 g were housed in individual cages with a 12-hour light–dark cycle and provided with tap water and a standard laboratory diet ad libitum before and after operation. All operations were performed under ether anaesthesia. This study was approved by the Animal Studies Committee of Oita Medical University, Japan and performed under the National Institutes of Health Standards of Animal Care.

*Received 24 November 1999*
*Revised 18 May 2000*
*Accepted 22 May 2000*

*Correspondence to:* S Goto

### Tumour cell line

The AH-130 rat ascites hepatoma cell line (a gift from Taiho Pharmaceutical Co., Ltd., Tokyo, Japan) was maintained by intra-peritoneal passage in male Donryu rats every 7–8 days. A suspension of the AH-130 tumour cells obtained from ascites was prepared in Hanks' balanced salts solution (HBSS) at a concentration of $5 \times 10^6$ cells/ml and 1 ml injected into the flank of Donryu rats. One week later, the subsequent subcutaneous solidified tumour was used for further experiments.

### Intra-hepatic tumour implantation

We have modified a technique of intra-hepatic tumour implantation (Yang et al, 1992). A brief outline of our modifications is as follows. A small cube about 1 $mm^3$ of tumour fragment was prepared by sharply mincing a 1-week-old vital outer zone subcutaneous growth of the tumour. The randomization followed the tumour implantation. A small incision on the median lobe of the liver was made using the tip of a No. 11 surgical blade, and the 1 $mm^3$ of tumour was gently inserted into the incision through an 18-gauge i.v. catheter (Terumo, Tokyo, Japan) with blunted tip. A small piece of Gelfoam (Upjohn, Tokyo, Japan) was used before and after the tumour insertion respectively. These modifications enabled us to bury the tumour fragment into the incision with haemostasis and without intra-peritoneal tumour cell leakage. Preliminary experiments have shown that a 1 $mm^3$ tumour fragment has reliably lodged in 100% of hepatic parenchyma five days after tumour implantation. This lodged tumour has grown to approximately 2 mm in diameter (data not shown). Based on this observation, 5 days following tumour implantation was used as the time for subsequent experiments.

### Experimental design

85 rats which underwent the intra-hepatic tumour implantation, were randomly allocated to the following three groups: sham operation group, 30% hepatectomy group, 60% hepatectomy group. Sham operations on controls consisted of laparotomy and gentle manipulation of the liver. 30% hepatectomy was performed by resecting the right lateral and caudate lobes of the liver. 60% hepatectomy included resection of the left lateral, right lateral, and caudate lobes of the liver by a modified method of Higgins and Anderson (1931). All animals following surgery received 10 ml normal saline solution intra-peritoneally for volume resuscitation and ceftriaxone sodium (Roche, Basel, Switzerland) 20 mg/body intramuscularly at the end of the procedure.

### Evaluation of tumour growth, body weight gain and liver regeneration rate

On post-operative day (POD) 7, ten rats from each group were weighed and then sacrificed after sampling whole blood from the inferior vena cava. After whole wet livers were harvested and weighed, the tumour and non-tumour portions were separated and weighed respectively. Moreover, the body weight gain and the liver regeneration rate were calculated.

### Evaluation for apoptosis

For liver sampling from each group on POD 7, non-tumour portions were obtained from the median lobe keeping approximately 1 cm away from tumour, and the tumour portion was dissected free from the normal hepatic parenchyma. These specimens were immersed in liquid nitrogen immediately after sampling and then stored at –80°C until analysed. For determination of DNA fragmentation on gel electrophoresis (Wyllie, 1980), the DNA ZOL® Reagent (GIBCO BRL, Grand Island, NY, USA) was used to extract the DNA. 20 μg of the isolated DNA was electrophoresed in a 2.0% agarose gel. The gel was stained with 0.5 μg/ml ethidium bromide for 15 min, destained with water for 1 hour and visualized under UV light. For TUNEL (terminal deoxynucleotidyl transferase (TdT)-mediated dUTP nick-end labelling) assay (Gavrieli et al, 1992) using the liver tissue on POD 7, liver specimens which were collected from 4 rats per each group, were formalin-fixed, paraffin-embedded and sectioned at 5 μm thickness. We used a DeadEnd™ Colorimetric Apoptosis Detection System (Promega Corporation, Madison, WI, USA) according to the manufacturer's instructions for the modified TUNEL assay. After staining with diaminobenzidine (DAB), the specimens were counterstained with haematoxylin, dehydrated and mounted. The apoptotic indices (AI) were calculated for each sample by counting the number of TUNEL-positive tumour cells divided by the total counted number of tumour cells. Approximately two thousand tumour cells were counted for each intra-hepatic tumour.

### Reverse transcriptase-polymerase chain reaction (RT-PCR)

RT-PCR was performed basically as previously described (Pan et al, 1999) using 0.1 μg of RNA. Reverse transcription of RNA from tissues, followed by PCR, was used to detect transforming growth factor-β1 (TGF-β1), tumour necrosis factor-α (TNF-α), Fas ligand (FasL) and Fas gene expression. The primers for all genes were designed according to the published rat complementary DNA (cDNA) sequences (Suda et al, 1993; Kimura et al, 1994; Farges et al, 1995). The PCR condition consisted of annealing at 55–60°C (55°C for β-actin, 56°C for TNF-α, 58°C for Fas/FasL and 60°C for TGF-β1) for 1 minute. PCR was performed at 25 cycles for β-actin, 30 cycles for Fas/FasL and 35 cycles for TGF-β1 and TNF-α. β-actin was amplified as an internal control to compare relative abundance of PCR products. The target bands were analysed densitometrically by using a GS-700 Imaging Densitometer (Bio-Rad, Hercules, CA, USA). All experiments were repeated more than twice.

### Quantitative determination of plasma TGF-β1 level

After sampling whole blood from the inferior vena cava pre-operatively and on PODs 1, 3 and 7, plasma was used for enzyme-linked immunosorbent assays (ELISA) for TGF-β1. The plasma TGF-β1 levels were determined using a PREDICTA TGF-β1 kit (Genzyme, Cambridge, MA, USA) for ELISA according to the manufacturer's instructions. As a strain control, sampling of 4 non-tumour bearing rats (naive animals) was also performed.

### Statistical analysis

All statistical analyses were performed with One-way ANOVA, and differences between groups were evaluated with Bonferroni multiple comparison. Data were expressed as the mean ± SD. The correlation between tumour weights and TGF-β1 levels was

*© 2000 Cancer Research Campaign*

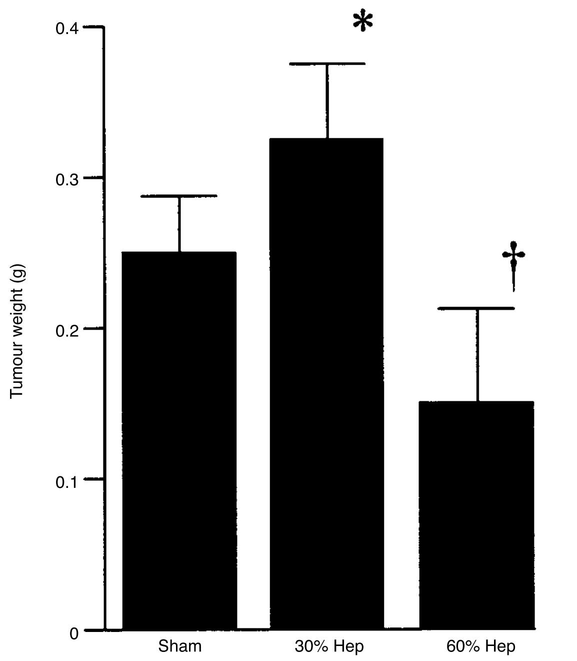

**Figure 1** Effects of hepatectomy on tumour growth. Hep, hepatectomy. Values represent mean ± SD, *n* = 10 per each group, **P* = 0.016 vs. sham, †*P* = 0.004 vs. sham obtained by simple regression analysis. These were performed using the SPSS statistical package (Chicago, IL, USA). *P* values of < 0.05 were considered to be statistically significant.

**Table 1.** Effects of operation on body weight and liver regeneration

| | Body weight gain | Liver regeneration rate (%) |
|---|---|---|
| Sham | 1.089±0.047 | 5.074±0.587 |
| 30% Hep | 1.060±0.034 | 4.842±0.393 |
| 60% Hep | 1.068±0.043 | 4.710±0.449 |

Autopsy (POD 7) body weight (g)/pre-operative body weight (g) was defined as the body weight gain. Autopsy (POD 7) liver weight (g)/pre-operative body weight (g) × 100 was defined as the liver regeneration rate (%). Hep: hepatectomy. Each value is expressed as the mean ± SD, n=10 per each group. No significant difference was observed in all groups. Statistics: one-way ANOVA

### RESULTS

### Tumour growth, body weight gain and liver regeneration rate

On POD 7, the intra-hepatic tumour weighed 0.245 ± 0.040 g (mean ± SD) in the sham. In 30% hepatectomy group, the tumour weight (0.321 ± 0.058 g) was significantly greater than that of the sham (*P* = 0.016), while the tumour (0.156 ± 0.067 g) in 60% hepatectomy group was significantly suppressed (*P* = 0.004 vs. sham) as shown in Figure 1. In all groups, no statistical significant difference was obtained in body weight gain on POD 7 (Table 1). The liver regeneration rate (%) in all groups was also similar, suggesting that DNA synthesis in the regenerating liver almost completed by restoration of liver volume by 1 week regardless of the percentage of hepatectomy.

### Apoptosis in intra-hepatic tumour

The DNA fragmentation with a ladder pattern was demonstrated in the tumour portion of three groups obtained on POD 7 (Figure 2A, lanes 2, 4 and 6). However, no laddering was observed in the DNA extracted from non-tumour portions in all groups (Figure 2A, lanes

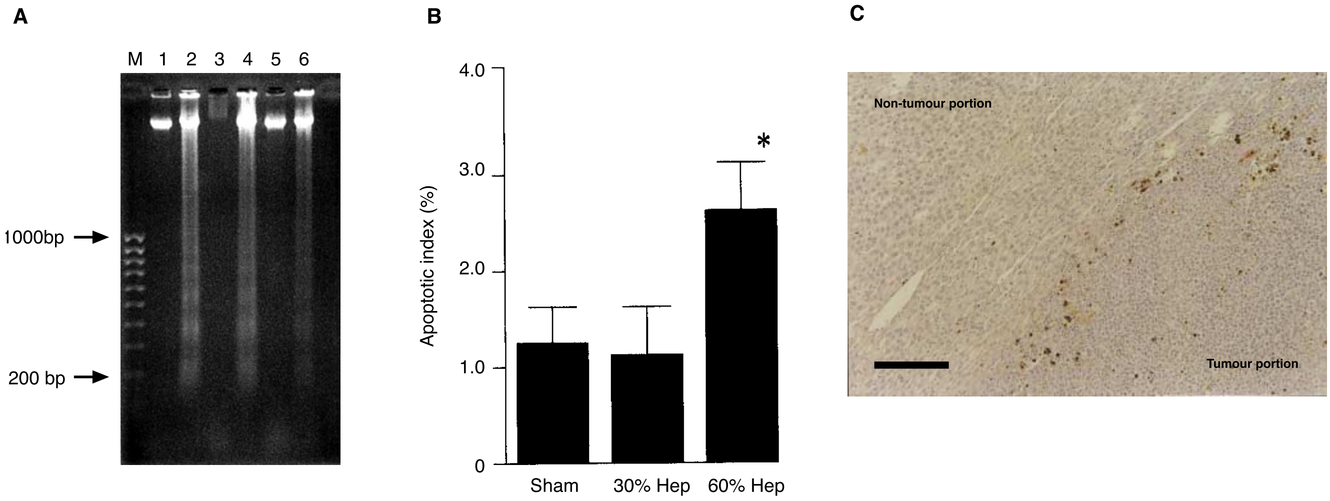

**Figure 2** (**A**) Hepatic DNA fragmentation on POD 7. Hep: hepatectomy. The lanes of the gel are as follows. The DNA molecular weight marker (lane M), non-tumour portion in 60% Hep (lane 1), tumour portion in 60% Hep (lane 2), non-tumour portion in 30% Hep (lane 3), tumour portion in 30% Hep (lane 4), non-tumour portion in sham (lane 5) and tumour portion in sham (lane 6). (**B**) TUNEL assay: Apoptotic indices (AI) in tumour portions. AI was calculated according to the formula described in the text. Hep: hepatectomy. Values represent mean ± SD. *n* = 4 per each group, **P* < 0.005 vs. 30% hepatectomy and sham. (**C**) TUNEL staining in 60% hepatectomy group. TUNEL-positive tumour cells were localized on the border between the tumour and non-tumour portion in 60% hepatectomy. Bar: 100 μm

© 2000 Cancer Research Campaign

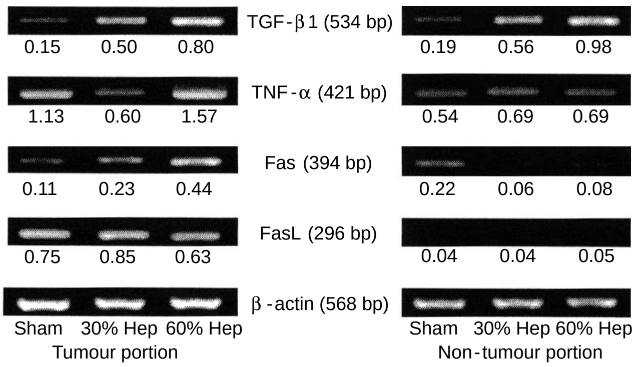

**Figure 3** Expression of mRNA in tumour and non-tumour portions. Hep, hepatectomy. The relative expression to β-actin, which was quantified by densitometric scanning, is shown at the bottom respectively as a ratio

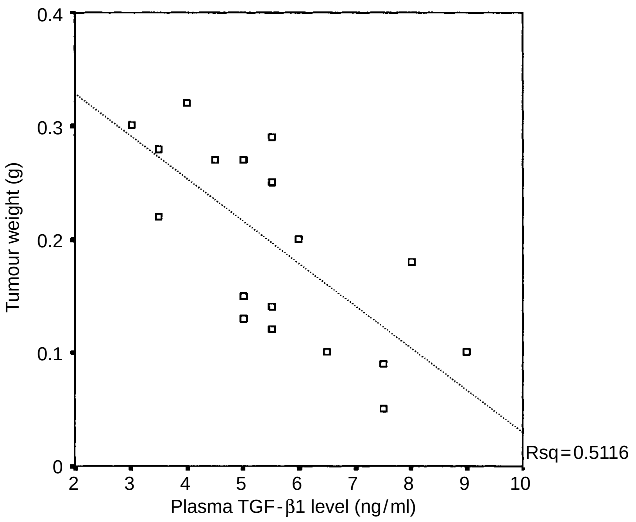

**Figure 4** Correlation between plasma TGF-β1 and tumour weight. Assays were performed in duplicate. Simple regression analysis, $n = 20$, $r = 0.715$, $P < 0.001$, Rsq, R square

1, 3 and 5). Using TUNEL assay with tumour portions from three groups, the apoptotic index (AI) in 60% hepatectomy was 2.58 ± 0.49% (mean ± SD), which was significantly higher than 1.08 ± 0.44% in 30% hepatectomy ($P = 0.002$) and 1.20 ± 0.31% in the sham ($P = 0.004$) as shown in Figure 2B. There was no significant difference of the AI between the 30% hepatectomy and sham group. The TUNEL-positive tumour cells were localized on the border between the tumour and non-tumour portion in 60% hepatectomy (Figure 2C).

### Expression of mRNA on POD 7

Expression of TGF-β1, TNF-α, FasL and Fas mRNA on POD 7 was analysed in the tumour and non-tumour portions of the liver of all groups as shown in Figure 3. β-actin expression was used as an internal control and the relative ratio of each gene expression to β-actin is shown. As for the tumour portion, TGF-β1, TNF-α and Fas mRNA in 60% hepatectomy exhibited higher expression levels than in the 30% hepatectomy and sham group. Moreover, Fas/FasL ratio (0.70) in 60% hepatectomy was much higher than the other two groups (0.15 in sham group and 0.27 in 30% hepatectomy). On the other hand, in non-tumour portion

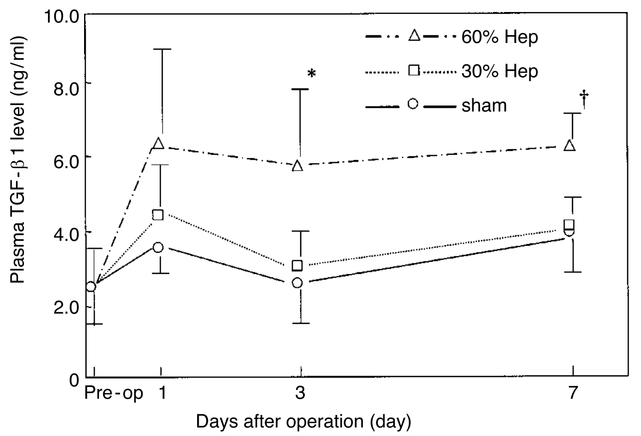

**Figure 5** TGF-β1 levels after the operation. Hep: hepatectomy. Assays were performed in duplicate. Values represent mean ± SD. *$P < 0.05$ vs. sham and 30% hepatectomy ($P$ value = 0.019 and 0.046, respectively), †$P < 0.005$ vs. sham and 30% hepatectomy ($P$ value = 0.002 and 0.004, respectively)

from 60% hepatectomy, the mRNA expression of TGF-β1 was higher than those in the 30% hepatectomy and sham group, while the mRNA levels of TNF-α did not show any significant difference among three groups. Non-tumour portions in three groups only faintly, if at all, expressed FasL mRNA although the mRNA expression of Fas was detectable only in sham group.

### Plasma TGF-β1 protein levels

On POD 7, intra-hepatic tumour weights were inversely correlated with the values of plasma TGF-β1 as demonstrated in Figure 4. A simple regression analysis demonstrated significant correlation between the TGF-β1 level and tumour weight ($n = 20$, $r = 0.715, P < 0.001$). Plasma TGF-β1 levels were measured pre-operatively and on PODs 1, 3 and 7 in all groups ($n$ = 4–5) respectively (Figure 5). TGF-β1 in 60% hepatectomy was elevated on POD 1 and maintained approximately 2-fold higher levels than in shams for the subsequent 6 days. On PODs 3 and 7, the values of TGF-β1 in 60% hepatectomy were 5.75 ± 1.94 ng/ml and 6.30 ± 0.76 ng/ml respectively, which were significantly higher than those in 30% hepatectomy (3.00 ± 0.71 and 4.10 ± 0.42) and sham (2.50 ± 0.91 and 3.90 ± 1.14). Pre-operatively, there were no significant differences in plasma TGF-β1 levels between tumour bearing rats and non-tumour bearing rats ($n$ = 4) for strain controls (data not shown).

### DISCUSSION

Little is known as to how hepatectomy is associated with the tumour growth of such established sites confined to the liver. Hepatic tumour models in rats generally require the exposure to a carcinogen (Yano et al, 1995), or the injection of a tumour cell suspension into the portal vein or into the sub-capsule of the liver, as previously reported (Mizutani et al, 1992; de Jong et al, 1995; Slooter et al, 1995; Picardo et al, 1998). However, since these procedures require a long time for tumour development or easily make a diffuse lodgement within the liver and peritoneal cavity, it is difficult to precisely evaluate the effect of hepatectomy on the established tumour growth in the remaining liver. We have established a reliable technique of intra-hepatic tumour implantation by modifying a technique reported by Yang et al (1992). Using this model, we demonstrated that the intra-hepatic tumours in 60% hepatectomy were significantly suppressed as compared with the sham, while the tumours in 30% hepatectomy were significantly accelerated.

Based on our TUNEL assay shown in Figure 2, one possible explanation for the above phenomenon is that 60% hepatectomy induced more apoptotic cell death of the remaining tumour than 30% hepatectomy or sham. The response of neoplastic cells to 'death signals' by far exceeds that of normal hepatocytes (Grasl-Kraupp et al, 1997). Additionally, certain death factors have also been defined as being capable of giving a death signal to induce apoptosis to tumour cells (Patel et al, 1998). In the present study, the mRNA expression of TGF-β1, TNFα and Fas/FasL in tumour portion was compared among groups as these molecules are involved in initiating the death signals associated with apoptosis in several types of cells including hepatomas (Lin and Chou, 1992; Owen-Schaub et al, 1994; Wong and Goeddel, 1994). The mRNA expression of TGF-β1, TNFα and Fas in the tumour portion of the 60% hepatectomy group was higher than that in the 30% hepatectomy group. It has also been reported that Fas and TGF-β1 act synergistically to induce cell death within tumours (Ashley et al, 1998). Additionally, in our experiments, plasma levels of TGF-β1 were inversely correlated with tumour weights on POD 7. It has therefore been strongly speculated that increased TGF-β1 in the 60% hepatectomy group may lead to cell death including Fas-mediated apoptosis for the remaining hepatic tumour.

The volume of the hepatic resection may also answer why plasma and mRNA levels of TGF-β1 in the 60% hepatectomy group were up-regulated compared with the 30% hepatectomy group. Our results demonstrated that restoration of hepatic volume occurred by 1 week in both groups subjected to 30% and 60% hepatic removal, suggesting that the speed or duration of hepatic regeneration was different between 60% hepatectomy and in 30% hepatectomy. This may also cause the differences in gene expression found in hepatocytes of non-tumour portions between 30% and 60% hepatectomy. In non-tumour portion from 60% hepatectomy, the gene expression of TGF-β1, which is involved in both reduced proliferation and apoptosis in hepatocytes, was higher than that in 30% hepatectomy. This is consistent with another report that expression of *c-myc* or *c-jun* mRNA, which are also involved in liver regeneration and apoptosis, increased in correlation to the amount of resected liver (Webber et al, 1994). Therefore, the hepatocytes in normal tissue adjacent to the tumour after 60% hepatectomy might express and secrete more apoptosis-related genes and proteins than 30% hepatectomy. This in turn resulted in the induction of tumour apoptosis by exposing the tumour to apoptotic inducers such as TGF-β1 (Fan et al, 1998). In the present study, plasma levels of TGF-β1 in 60% hepatectomy were significantly higher than those in 30% hepatectomy. This may also suggest a systemic effect of hepatectomy on the growth of tumour implanted in extra-hepatic sites (Ono et al, 1986). However, further studies including the possible effect of hepatocyte and vascular endothelial growth factors on tumour growth after varying degrees of hepatic resection are required.

Several reports have also demonstrated that liver regeneration after hepatic resection might induce a host immune response to the tumour (Ono et al, 1986; Doerr et al, 1989). Non-parenchymal cells such as Kupffer cells and pit cells (hepatic natural killer cells), or other lymphocytes mainly exhibit inhibitory effects on tumour cells for cytotoxicity (Bayon et al, 1996; Vermijlen et al, 1999). These might have been extremely activated during regeneration after major hepatic resection, and it is these activated cells that may be responsible for the increased TNFα mRNA expression on the tumour portion of the 60% hepatectomy group in our present experiments. Moreover, previous studies have described that tumour cells can escape from host immune attack through the down-regulation of Fas receptors and the up-regulation of FasL expression on the tumour site for the development of the malignant tumour (Higaki et al, 1996; Strand et al, 1996). However, in our experiments, Fas/FasL ratio in 60% hepatectomy was much higher than those in the sham and 30% hepatectomy group. These results suggest that the tumour in 60% hepatectomy animals might have easily suffered from host immune attack including cytotoxic T lymphocytes and natural killer cells through Fas-induced apoptosis.

In conclusion, we have demonstrated that major hepatic resection might suppress the growth of tumours remaining in the residual liver. These results may indicate that the extended hepatic resection for primary HCC is recommended although the choice of type of liver resection should be based on a balance between the need for preserving liver function and indications for radical resection of the tumour. Further studies, including other apoptosis-related receptors, are underway to elucidate the mechanisms of tumour apoptosis induced by major hepatic resection.

### ACKNOWLEDGEMENTS

We thank Taiho Pharmaceutical Co., Ltd. for providing AH-130 cells, and Dr Yuji Morii (Department of Surgery I, Oita Medical University, Japan) for helpful suggestions and technical assistance. We are also grateful to Dr Roger Lord (Department of Cardiology, University of Wales College of Medicine, UK) and Mr John Ash (Biomembrane Co., Ltd., Australia) for critical evaluation of this manuscript.

### REFERENCES

Ashley DM, Kong FM, Bigner DD and Hale LP (1998) Endogenous expression of transforming growth factor beta1 inhibits growth and tumorigenicity and enhances Fas-mediated apoptosis in a murine high-grade glioma model. *Cancer Res* **58**: 302–309

Bayon LG, Izquierdo MA, Sirovich I, van Rooijen N, Beelen RH and Meijer S (1996) Role of Kupffer cells in arresting circulating tumor cells and controlling metastatic growth in the liver. *Hepatology* **23**: 1224–1231

Castillo MH, Doerr RJ, Paolini N Jr, Cohen S and Goldrosen M (1989) Hepatectomy prolongs survival of mice with induced liver metastases. *Arch Surg* **124**: 167–169

de Jong KP, Lont HE, Bijma AM, Brouwers MA, de Vries EG, van Veen ML, Marquet RL, Slooff MJ and Terpstra OT (1995) The effect of partial hepatectomy on tumor growth in rats: in vivo and in vitro studies. *Hepatology* **22**: 1263–1272

Doerr R, Castillo M, Evans P, Paolini N, Goldrosen M and Cohen SA (1989) Partial hepatectomy augments the liver's antitumor response. *Arch Surg* **124**: 170–174

Ezaki T, Yukaya H, Ogawa Y, Chang YC and Nagasue N (1989) Recurrent form of hepatocellular carcinoma after partial hepatic resection. *Hepatogastroenterology* **36**: 164–167

Fan G, Kren BT and Steer CJ (1998) Regulation of apoptosis-associated genes in the regenerating liver. *Semin Liver Dis* **18**: 123–140

Farges O, Morris PJ and Dallman MJ (1995) Spontaneous acceptance of rat liver allografts is associated with an early downregulation of intragraft interleukin-4 messenger RNA expression. *Hepatology* **21**: 767–775

Gavrieli Y, Sherman Y and Ben-Sasson SA (1992) Identification of programmed cell death in situ via specific labeling of nuclear DNA fragmentation. *J Cell Biol* **119**: 493–501

Grasl-Kraupp B, Ruttkay-Nedecky B, Mullauer L, Taper H, Huber W, Bursch W and Schulte-Hermann R (1997) Inherent increase of apoptosis in liver tumors:

 *© 2000 Cancer Research Campaign*

implications for carcinogenesis and tumor regression. *Hepatology* **25**: 906–912

Higaki K, Yano H and Kojiro M (1996) Fas antigen expression and its relationship with apoptosis in human hepatocellular carcinoma and noncancerous tissues. *Am J Pathol* **149**: 429–437

Higgins GM and Anderson RM (1931) Experimental pathology of the liver; restoration of the liver of the white rat following partial surgical removal. *Arch Pathol* **12**: 186–202

Kimura K, Wakatsuki T and Yamamoto M (1994) A variant mRNA species encoding a truncated form of Fas antigen in the rat liver. *Biochem Biophys Res Commun* **198**: 666–674

Kosuge T, Makuuchi M, Takayama T, Yamamoto J, Shimada K and Yamasaki S (1993) Long-term results after resection of hepatocellular carcinoma: experience of 480 cases. *Hepatogastroenterology* **40**: 328–332

Lin JK and Chou CK (1992) In vitro apoptosis in the human hepatoma cell line induced by transforming growth factor beta 1. *Cancer Res* **52**: 385–388

Mizutani J, Hiraoka T, Yamashita R and Miyauchi Y (1992) Promotion of hepatic metastases by liver resection in the rat. *Br J Cancer* **65**: 794–797

Nagao T, Inoue S, Yoshimi F, Sodeyama M, Omori Y, Mizuta T, Kawano N and Morioka Y (1990) Postoperative recurrence of hepatocellular carcinoma. *Ann Surg* **211**: 28–33

Ono M, Tanaka N and Orita K (1986) Complete regression of mouse hepatoma transplanted after partial hepatectomy and the immunological mechanism of such regression. *Cancer Res* **46**: 5049–5053

Owen-Schaub LB, Radinsky R, Kruzel E, Berry K and Yonehara S (1994) Anti-Fas on nonhematopoietic tumors: levels of Fas/APO-1 and bcl-2 are not predictive of biological responsiveness. *Cancer Res* **54**: 1580–1586

Pan TL, Goto S, Lin YC, Lord R, Chiang KC, Lai CY, Chen YS, Eng HL, Cheng YF, Tatsuma T, Kitano S, Lin CL and Chen CL (1999) The Fas and Fas ligand pathways in liver allograft tolerance. *Clin Exp Immunol* **118**: 180–187

Patel T, Roberts LR, Jones BA and Gores GJ (1998) Dysregulation of apoptosis as a mechanism of liver disease: an overview. *Semin Liver Dis* **18**: 105–114

Picardo A, Karpoff HM, Ng B, Lee J, Brennan MF and Fong Y (1998) Partial hepatectomy accelerates local tumor growth: potential roles of local cytokine activation. *Surgery* **124**: 57–64

Shimada M, Takenaka K, Taguchi K, Fujiwara Y, Gion T, Kajiyama K, Maeda T, Shirabe K, Yanaga K and Sugimachi K (1998) Prognostic factors after repeat hepatectomy for recurrent hepatocellular carcinoma. *Ann Surg* **227**: 80–85

Slooter GD, Marquet RL, Jeekel J and Ijzermans JN (1995) Tumour growth stimulation after partial hepatectomy can be reduced by treatment with tumour necrosis factor alpha. *Br J Surg* **82**: 129–132

Strand S, Hofmann WJ, Hug H, Muller M, Otto G, Strand D, Mariani SM, Stremmel W, Krammer PH and Galle PR (1996) Lymphocyte apoptosis induced by CD95 (APO-1/Fas) ligand-expressing tumor cells – a mechanism of immune evasion? *Nat Med* **2**: 1361–1366

Suda T, Takahashi T, Golstein P and Nagata S (1993) Molecular cloning and expression of the Fas ligand, a novel member of the tumor necrosis factor family. *Cell* **75**: 1169–1178

Utsunomiya T, Matsumata T, Adachi E, Honda H and Sugimachi K (1992) Limitations of current preoperative liver imaging techniques for intrahepatic metastatic nodules of hepatocellular carcinoma. *Hepatology* **16**: 694–701

Vermijlen D, Luo D, Robaye B, Seynaeve C, Baekeland M and Wisse E (1999) Pit cells (Hepatic natural killer cells) of the rat induce apoptosis in colon carcinoma cells by the perforin/granzyme pathway. *Hepatology* **29**: 51–56

Webber EM, Godowski PJ and Fausto N (1994) In vivo response of hepatocytes to growth factors requires an initial priming stimulus. *Hepatology* **19**: 489–497

Wong GH and Goeddel DV (1994) Fas antigen and p55 TNF receptor signal apoptosis through distinct pathways. *J Immunol* **152**: 1751–1755

Wyllie AH (1980) Glucocorticoid-induced thymocyte apoptosis is associated with endogenous endonuclease activation. *Nature* **284**: 555–556

Yamamoto J, Kosuge T, Takayama T, Shimada K, Yamasaki S, Ozaki H, Yamaguchi N and Makuuchi M (1996) Recurrence of hepatocellular carcinoma after surgery. *Br J Surg* **83**: 1219–1222

Yang R, Rescorla FJ, Reilly CR, Faught PR, Sanghvi NT, Lumeng L, Franklin TD Jr and Grosfeld JL (1992) A reproducible rat liver cancer model for experimental therapy: introducing a technique of intrahepatic tumor implantation. *J Surg Res* **52**: 193–198

Yano K, Fukuda Y, Sumimoto R, Ito H, Asahara T and Dohi K (1995) Development of a rat model for orthotopic liver transplantation for hepatocellular carcinoma. *Surgery* **118**: 539–546

*© 2000 Cancer Research Campaign*

## References

[PDF_00463] Ashley D. M., Kong F. M., Bigner D. D., Hale L. P. (1998). Endogenous expression of transforming growth factor beta1 inhibits growth and tumorigenicity and enhances Fas-mediated apoptosis in a murine high-grade glioma model.. Cancer Res.

[PDF_00467] Bayón L. G., Izquierdo M. A., Sirovich I., van Rooijen N., Beelen R. H., Meijer S. (1996). Role of Kupffer cells in arresting circulating tumor cells and controlling metastatic growth in the liver.. Hepatology.

[PDF_00470] Castillo M. H., Doerr R. J., Paolini N., Cohen S., Goldrosen M. (1989). Hepatectomy prolongs survival of mice with induced liver metastases.. Arch Surg.

[PDF_00477] Doerr R., Castillo M., Evans P., Paolini N., Goldrosen M., Cohen S. A. (1989). Partial hepatectomy augments the liver's antitumor response.. Arch Surg.

[PDF_00479] Ezaki T., Yukaya H., Ogawa Y., Chang Y. C., Nagasue N. (1989). Recurrent form of hepatocellular carcinoma after partial hepatic resection.. Hepatogastroenterology.

[PDF_00482] Fan G., Kren B. T., Steer C. J. (1998). Regulation of apoptosis-associated genes in the regenerating liver.. Semin Liver Dis.

[PDF_00484] Farges O., Morris P. J., Dallman M. J. (1995). Spontaneous acceptance of rat liver allografts is associated with an early downregulation of intragraft interleukin-4 messenger RNA expression.. Hepatology.

[PDF_00487] Gavrieli Y., Sherman Y., Ben-Sasson S. A. (1992). Identification of programmed cell death in situ via specific labeling of nuclear DNA fragmentation.. J Cell Biol.

[PDF_00490] Grasl-Kraupp B., Ruttkay-Nedecky B., Müllauer L., Taper H., Huber W., Bursch W., Schulte-Hermann R. (1997). Inherent increase of apoptosis in liver tumors: implications for carcinogenesis and tumor regression.. Hepatology.

[PDF_00496] Higaki K., Yano H., Kojiro M. (1996). Fas antigen expression and its relationship with apoptosis in human hepatocellular carcinoma and noncancerous tissues.. Am J Pathol.

[PDF_00502] Kimura K., Wakatsuki T., Yamamoto M. (1994). A variant mRNA species encoding a truncated form of Fas antigen in the rat liver.. Biochem Biophys Res Commun.

[PDF_00505] Kosuge T., Makuuchi M., Takayama T., Yamamoto J., Shimada K., Yamasaki S. (1993). Long-term results after resection of hepatocellular carcinoma: experience of 480 cases.. Hepatogastroenterology.

[PDF_00508] Lin J. K., Chou C. K. (1992). In vitro apoptosis in the human hepatoma cell line induced by transforming growth factor beta 1.. Cancer Res.

[PDF_00510] Mizutani J., Hiraoka T., Yamashita R., Miyauchi Y. (1992). Promotion of hepatic metastases by liver resection in the rat.. Br J Cancer.

[PDF_00512] Nagao T., Inoue S., Yoshimi F., Sodeyama M., Omori Y., Mizuta T., Kawano N., Morioka Y. (1990). Postoperative recurrence of hepatocellular carcinoma.. Ann Surg.

[PDF_00515] Ono M., Tanaka N., Orita K. (1986). Complete regression of mouse hepatoma transplanted after partial hepatectomy and the immunological mechanism of such regression.. Cancer Res.

[PDF_00518] Owen-Schaub L. B., Radinsky R., Kruzel E., Berry K., Yonehara S. (1994). Anti-Fas on nonhematopoietic tumors: levels of Fas/APO-1 and bcl-2 are not predictive of biological responsiveness.. Cancer Res.

[PDF_00521] Pan T. L., Goto S., Lin Y. C., Lord R., Chiang K. C., Lai C. Y., Chen Y. S., Eng H. L., Cheng Y. F., Tatsuma T. (1999). The fas and fas ligand pathways in liver allograft tolerance.. Clin Exp Immunol.

[PDF_00524] Patel T., Roberts L. R., Jones B. A., Gores G. J. (1998). Dysregulation of apoptosis as a mechanism of liver disease: an overview.. Semin Liver Dis.

[PDF_00526] Picardo A., Karpoff H. M., Ng B., Lee J., Brennan M. F., Fong Y. (1998). Partial hepatectomy accelerates local tumor growth: potential roles of local cytokine activation.. Surgery.

[PDF_00529] Shimada M., Takenaka K., Taguchi K., Fujiwara Y., Gion T., Kajiyama K., Maeda T., Shirabe K., Yanaga K., Sugimachi K. (1998). Prognostic factors after repeat hepatectomy for recurrent hepatocellular carcinoma.. Ann Surg.

[PDF_00532] Slooter G. D., Marquet R. L., Jeekel J., Ijzermans J. N. (1995). Tumour growth stimulation after partial hepatectomy can be reduced by treatment with tumour necrosis factor alpha.. Br J Surg.

[PDF_00535] Strand S., Hofmann W. J., Hug H., Müller M., Otto G., Strand D., Mariani S. M., Stremmel W., Krammer P. H., Galle P. R. (1996). Lymphocyte apoptosis induced by CD95 (APO-1/Fas) ligand-expressing tumor cells--a mechanism of immune evasion?. Nat Med.

[PDF_00539] Suda T., Takahashi T., Golstein P., Nagata S. (1993). Molecular cloning and expression of the Fas ligand, a novel member of the tumor necrosis factor family.. Cell.

[PDF_00542] Utsunomiya T., Matsumata T., Adachi E., Honda H., Sugimachi K. (1992). Limitations of current preoperative liver imaging techniques for intrahepatic metastatic nodules of hepatocellular carcinoma.. Hepatology.

[PDF_00545] Vermijlen D., Luo D., Robaye B., Seynaeve C., Baekeland M., Wisse E. (1999). Pit cells (Hepatic natural killer cells) of the rat induce apoptosis in colon carcinoma cells by the perforin/granzyme pathway.. Hepatology.

[PDF_00548] Webber E. M., Godowski P. J., Fausto N. (1994). In vivo response of hepatocytes to growth factors requires an initial priming stimulus.. Hepatology.

[PDF_00550] Wong G. H., Goeddel D. V. (1994). Fas antigen and p55 TNF receptor signal apoptosis through distinct pathways.. J Immunol.

[PDF_00552] Wyllie A. H. (1980). Glucocorticoid-induced thymocyte apoptosis is associated with endogenous endonuclease activation.. Nature.

[PDF_00554] Yamamoto J., Kosuge T., Takayama T., Shimada K., Yamasaki S., Ozaki H., Yamaguchi N., Makuuchi M. (1996). Recurrence of hepatocellular carcinoma after surgery.. Br J Surg.

[PDF_00557] Yang R., Rescorla F. J., Reilly C. R., Faught P. R., Sanghvi N. T., Lumeng L., Franklin T. D., Grosfeld J. L. (1992). A reproducible rat liver cancer model for experimental therapy: introducing a technique of intrahepatic tumor implantation.. J Surg Res.

[PDF_00561] Yano K., Fukuda Y., Sumimoto R., Ito H., Asahara T., Dohi K. (1995). Development of a rat model for orthotopic liver transplantation for hepatocellular carcinoma.. Surgery.

[PDF_00473] de Jong K. P., Lont H. E., Bijma A. M., Brouwers M. A., de Vries E. G., van Veen M. L., Marquet R. L., Slooff M. J., Terpstra O. T. (1995). The effect of partial hepatectomy on tumor growth in rats: in vivo and in vitro studies.. Hepatology.

